# Effects of aging on functional connectivity in a neurodegenerative risk cohort: resting state versus task measurement using near-infrared spectroscopy

**DOI:** 10.1038/s41598-022-13326-7

**Published:** 2022-07-04

**Authors:** Leonore Blum, Anna Hofmann, David Rosenbaum, Morad Elshehabi, Ulrike Suenkel, Andreas J. Fallgatter, Ann-Christine Ehlis, Florian G. Metzger

**Affiliations:** 1 Department of Psychiatry and Psychotherapy, Tübingen Center for Mental Health (TüCMH), University Hospital of Tuebingen, Tuebingen, Germany; 2 German Center for Neurodegenerative Diseases (DZNE), University of Tuebingen, Tuebingen, Germany; 3 Geriatric Center, University Hospital of Tuebingen, Tuebingen, Germany; 4 LEAD Graduate School & Research Network, University of Tuebingen, Tuebingen, Germany; 5 Vitos Hospital of Psychiatry and Psychotherapy Haina, Haina, Germany; 6 Hertie Institute for Clinical Brain Research, Tuebingen, Germany; 7 Department of Neurology and Neurodegenerative Diseases, University Hospital Tuebingen, Hoppe-Seyler-Str. 3, 72076 Tuebingen, Germany; 8 Department of Neurology, University Hospitals of Schleswig-Holstein, Campus-Kiel, Kiel, Germany

**Keywords:** Neuroscience, Psychology

## Abstract

Changes in functional brain organization are considered to be particularly sensitive to age-related effects and may precede structural cognitive decline. Recent research focuses on aging processes determined by resting state (RS) functional connectivity (FC), but little is known about differences in FC during RS and cognitive task conditions in elderly participants. The purpose of this study is to compare FC within and between the cognitive control (CCN) and dorsal attention network (DAN) at RS and during a cognitive task using functional near-infrared spectroscopy (fNIRS). In a matched, neurodegenerative high-risk cohort comprising early (n = 98; 50–65 y) and late (n = 98; 65–85 y) elder subjects, FC was measured at RS and during performance of the Trail Making Test (TMT) via fNIRS. Both, under RS and task conditions our results revealed a main effect for age, characterized by reduced FC for late elder subjects within the left inferior frontal gyrus. During performance of the TMT, negative correlations of age and FC were confirmed in various regions of the CCN and DAN. For the whole sample, FC of within-region connections was elevated, while FC between regions was decreased at RS. The results confirm a reorganization of functional brain connectivity with increasing age and cognitive demands.

## Introduction

It is well known that aging is associated with neurodegeneration. However, natural aging processes are still far from being comprehensively understood. A major challenge lies in the distinction between physiological and pathological aging, since the brain changes its structure and thus also its function during the entire lifetime^1,2^. At the cognitive level, aging is associated with a decrease in executive functions, e.g. processing speed, working memory and mental flexibility^3^. On a neuropathological level, tissue reduction of grey and white matter^4–11^, loss of synaptic connections^12^ and amyloid deposition in non-demented individuals^13^ have been observed in old age. Accordingly, an essential focus of research is the identification of correlations between cognitive and structural neuronal changes. In addition to anatomical remodeling, a shift of functional connections in old age has also been described, e.g. various patterns of hypo- or hyper-recruitment of brain regions have been observed during the senium^14–17^. It was even hypothesized that functional changes precede the structural reorganization of the brain^18^. Changes in functional brain organization are therefore considered to be particularly sensitive to early age-related effects. First described by Friston et al.^19^, functional connectivity (FC) is defined as the temporal relationship between spatially separated neurophysiological processes. These functional connections between different brain areas exist both at rest and during task accomplishment and are considered as functional networks such as the cognitive control network (CCN), dorsal attention network (DAN), default mode network (DMN) and salience network (SN)^20^. In the past, it has already been shown that FC differs between subjects with mild cognitive impairment (MCI) or Alzheimer's disease (AD) and healthy controls^21–24^. Within the clinical context, a malfunctioning of especially the CCN and DAN has been connected to psychiatric disorders such as depression^25,26^ as well as Alzheimer’s Dementia^27^. In addition, there is evidence for changes in the FC pattern with increasing age. The consensus of aging studies indicates a decrease in FC within resting state (RS) networks such as the default mode network (DMN) or the salience network (SN)^28–31^, concomitant with an increase in FC between the different networks^28,32,33^ as well as a general association of aging with reduced global efficiency and modularity^34^. Moreover, Esposito et al.^35^ described a reduction of the physiological anticorrelation activity between the DMN and the DAN in RS as part of a normal aging process and MCI as a status in which these changes are even more pronounced. Similar observations have on principle also been made during different tasks^36^. However, in most studies on aging processes, FC has been determined by RS measurements ^28,32,37–39^ as this is easier to implement in clinical experimental setups.

Indeed, we hypothesized that deficits associated with higher age as well as early stages of neurodegeneration, for which age actually is the main risk factor, would initially show up as reduced performance and/or altered neural functionality in the management of cognitive tasks. Therefore, we compared the measurement of FC via functional near-infrared spectroscopy (fNIRS) during resting state (rsFC) with a measurement during execution of the Trail Making Test (TMTFC), a neuropsychological task for the assessment of executive (i.e., frontal lobe) functions^37^.

In particular the TMT-B subtest requires activation of frontal cortical structures such as the inferior and middle frontal cortex as well as the dorsolateral prefrontal cortex (DLPFC)^2,39^. Performing the TMT therefore induces an activation of the CCN as well as the DAN, as it activates the dorsal parts of the lateral prefrontal cortex (DLPFC), cingulate cortex (dACC = dorsal anterior cingulate cortex) and parietal cortex/somatosensory association cortex^40–42^. We chose fNIRS as it not only exhibits the particular advantage of being applicable to participants sitting at a desk in an upright position to perform the task (including hand/arm movements) under natural conditions^38^, but also because it is known to reliably reflect cortical activity in the above named areas in an elderly cohort^26,43^.

The study at hand thus aims to improve our understanding of FC during task completion in contrast to RS measurements for the purpose of early detection of age-related changes indicative of incipient neurodegenerative processes. To this end, we investigated two groups of elderly participants, early (50 to 65 years of age) versus late elders (65 to 85 years of age) enriched with, but also matched for other neurodegenerative risk factors like REM sleep behavior disorder (RBD) or depression. The rationale behind the chosen cut-off at 65 years was the following. If an individual develops a neurodegenerative dementia earlier than 65 years of age, the particular diagnosis (e.g. AD) is indicated as *early-onset* and the etiological background (e.g. genetic factors) is rather complex in many cases^44^. After the age of 65 years, the development of a neurodegenerative disorder like AD becomes more and more frequent and is therefore in most cases considered as senile. Even within the current version of the International Classification of Diseases by the World Health Organization (WHO), this diagnostic cut-off at the age of 65 years is used^45^. Our first goal was to determine the performance of the late elder participants in comparison to the early elder group based on the number of items completed during TMT execution. Secondly, potential differences should be investigated between rsFC and TMTFC, again within the late compared to the early elder subgroup. In this regard, we were especially interested in the FC patterns during TMT-B execution in contrast to RS since this subtask is considered most demanding and therefore might serve as a sensitive marker for subtle cognitive decline. In general, we assumed the early elder subjects to perform worse in the TMT compared to the early elders. Irrespective of age, we presumed that FC would be higher during task completion than at rest.

## Participants and methods

### Study population

The participants originated from the *Tuebinger evaluation of risk factors for early detection of neurodegeneration* (TREND)-study database. This is a large-scale study from the Department of Neurology and the Department of Psychiatry and Psychotherapy of the University Hospital of Tuebingen, Germany, initiated in 2009, which aims at investigating possible prodromal markers for neurodegenerative diseases^46–48^. The study was approved by the Ethics Committee of the University of Tuebingen and is in accordance with the standards of the World Medical Association's Declaration of Helsinki. Informed consent was obtained from all participants included in this study. The TREND study is conducted via biennial assessments. Inclusion and exclusion criteria initially were as follows: age between 50 and 80 years, no neurodegenerative disease at baseline and—if applicable—at least one of the following prodromal markers for neurodegeneration: depression, hyposmia and RBD, characterized by loss of physiological atonia during REM sleep. Especially individuals suffering from the latter have a risk of about 50% to develop Parkinson’s Disease or dementia within ten years^49^. Individuals who did not experience any of these symptoms were recruited as controls which concerns nearly half of the participants. The assessment battery includes medical history, neurological examination, transcranial sonography, olfactory, autonomic and cognitive testing with the CERADplus battery (Consortium to Establish a Registry for Alzheimer’s Disease)^50^ and MOCA (Montreal Cognitive Assessment)^51^ as well as self-report questionnaires assessing RBD, mood (Beck Depression Inventory, version I) as well as quality of life (for more details visit: https://www.trend-studie.de/). Study data are collected and managed using REDCap electronic data capture tools hosted at the University of Tuebingen^52^.

In the study at hand, a subsample from the TREND cohort of in total n = 196 participants (50–85 years of age) has been investigated, comprising n = 98 late (> 65 years of age) as well as n = 98 early elders (< 65 years of age), matched according to education, gender, and risk-factors for neurodegenerative diseases and/or cognitive decline (amnestic MCI (aMCI) and RBD). The early elder group consisted of 54% female participants, had a mean age of 60.23 years (*SD* = 2.98) and a mean education of 14.23 years (*SD* = 2.44). Within the late elder cohort, 43% of participants were female, the mean age was 70.27 years (*SD* = 4.46) with on average 13.96 years (*SD* = 2.69) of education. Due to the matching procedure, the early and late elder subgroup did not differ regarding the frequency of neurodegenerative risk factors (aMCI: χ^2^(1) = 1.71, *p* = 0.19, RBD: χ^2^(1) = 0.02, *p* = 0.89), years of education (*t*(194) = 0.75, *p* = 0.453, *d* = 0.11) or gender (χ^2^(1) = 2.47, *p* = 0.116). 87% of the participants were on medication, in particular blood pressure medication (41%), anticoagulants (20%) and antidepressants (11%). The early and late elder sample did not differ in terms of their medication status (χ^2^(1, *n* = 196) = 3.71, *p* = 0.054) (Table 1).

**Table 1:** Epidemiological data of the investigated cohort.

Characteristics	Early elders	Late elders	Statistics
Age (mean years)	60.23 (SD = 2.98)	70.27 (SD = 4.46)	–
Gender (% female)	54.10	42.90	*p* = 0.11
Education (mean years)	14.23 (SD = 2.44)	13.96 (SD = 2.69)	*p* = 0.45
aMCI (% diagnosed)	9.20	15.30	*p* = 0.19
RBD (% diagnosed)	46.90	48.00	*p* = 0.89
Medication (% intake)	82.70	91.80	*p* = 0.05
MMSE* (mean score)	28.47 (SD = 1.46)	28.25 (SD = 1.57)	*p* = 0.31
BDI** (mean score)	7.84 (SD = 9.39)	8.02 (SD = 8.80)	*p* = 0.88
Global cognition*** (CERAD mean total score)	88.24 (SD = 5.94)	81.79 (SD = 8.73)	*p* < 0.001
Episodic memory**** (WMS-IV, I/II percentile rank)	55.68/59.89	42.32/57.99	*p* = 0.003/*p* = 0.67

aMCI, amnestic mild cognitive impairment; RBD, rapid eye movement sleep behavior disorder; SD, standard deviation.

*Mini Mental State Examination (MMSE).

**depressive characteristics according to Beck's Depression Inventory (BDI-I).

***Consortium to Establish a Registry for Alzheimer’s Disease (CERAD) total score^53^.

****Logical Memory (LM) subtest of the Wechsler Memory Scale-IV (WMS-IV), I = direct recall, II = delayed recall).

### Trail Making Test

The TMT as a subtask of the CERAD-Plus test battery^50^ is a standardized neuropsychological test procedure for the detection of cognitive deficits, especially checking for executive functions, working memory and mental flexibility^37^. The modified version of the TMT used in our study consists of three sub-tests: TMT-A, TMT-B and a so-called TMT-C. Each of the sheets contains 25 items. During the TMT-A, subjects are instructed to link randomly distributed numbers in an ascending order as quickly as possible. In the TMT-B, numbers and letters must be connected alternately according to the ascending number chain and alphabet. In addition to motor speed, visual search function and working memory, the TMT-B also tests the ability of task-switching. The TMT-C was developed as a control condition for functional imaging to simply assess motor activity by tracing pre-drawn lines. In order to minimize environmental influences, the measurement took place in a quiet, darkened room. The participants were instructed to sit upright and avoid head movements during the simultaneous fNIRS measurement. Physiological working posture was ensured by an inclined desk. The TMT was performed in a block design as follows: TMT-A–TMT-B–TMT-C–TMT-A–TMT-B–TMT-C–TMT-A–TMT-B. Blocks were separated by a 30-s pause. Implementation started with a five-minutes resting measurement with closed eyes, then the first two blocks were conducted. After a short instruction and exercise, the test was performed without a time limit to ensure a later standardization of analysis for the TMT behavioral data. During the subsequent six blocks, the completion time was limited to 30 s. The number of items and errors was documented for each participant.

### fNIRS and preprocessing of data

**Figure 1:**
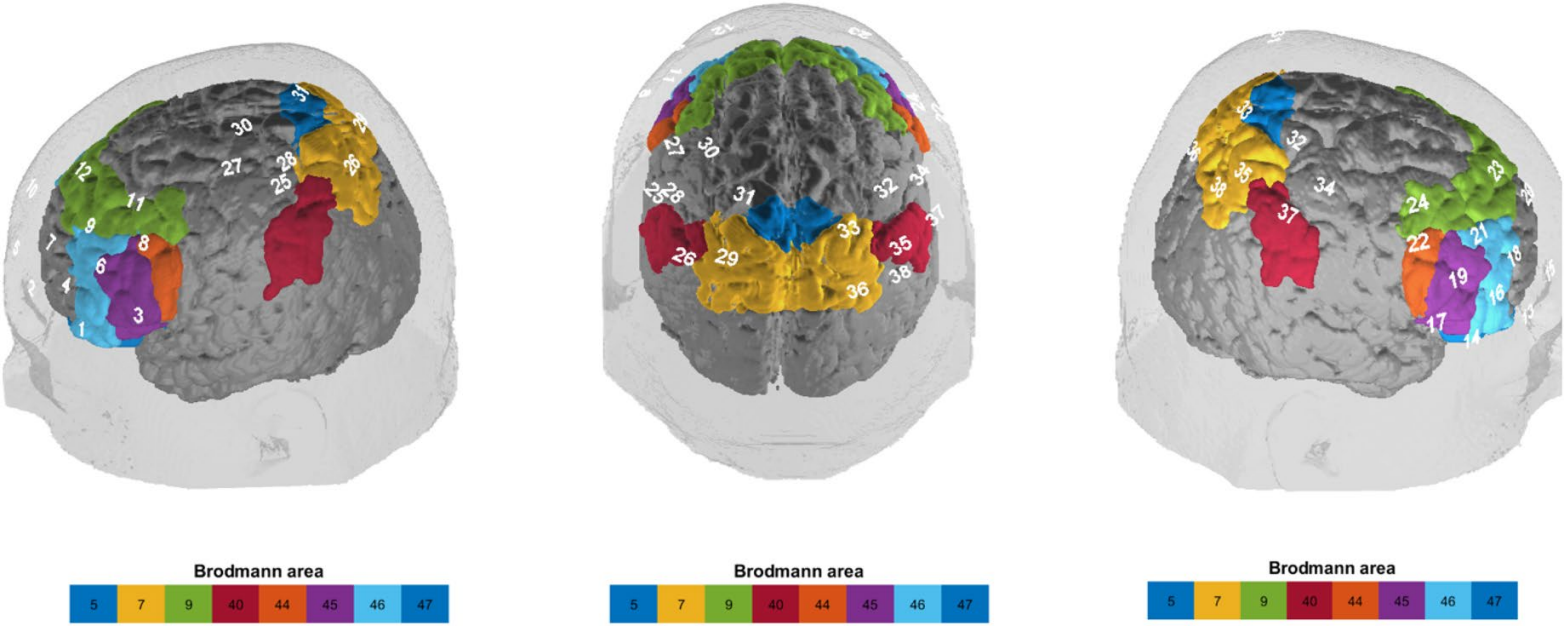
fNIRS: the white numbers above on the cortical surface represent the channel denotation, whereas the black numbers below within the colored boxes correspond to the indicated Brodmann areas (left—back—right view).

The concentration of oxygenated hemoglobin (O2Hb) during brain activation was measured by a continuous wave, multichannel fNIRS system with a sampling rate of 10 Hz. A total of 38 channels were measured (24 fronto-temporal, 14 parietal; ﻿Fig. 1) with fixed inter-optode distances of 30 mm (no short-distance channels were included). Exact anatomical fixation was performed using an optode holder cap with reference points F3/F4 and Fp1/Fp2 (fronto-temporal) and C3/C4 (parietal)^54^. Corresponding brain areas of each channel were extrapolated from reference points as in the work by Singh et al.^55^ as well as by other colleagues ^56,57^ based on the Colin 27 template. Data were recorded with a semiconductor laser and avalanche diodes at two wavelengths (695 ± 20 and 830 ± 20 nm) with 4.0 ± 0.2 mW for each wavelength at each optode. The acquisition and pre-processing of the measurement data was performed with the software of the ETG-4000 (ETG-4000 Optical Topography System; Hitachi Medical Co., Japan).

Data were assessed during the 5-min RS measurement and under subsequent TMT performance. We ensured proper time-locking between fNIRS acquisition and the TMT task by the investigator pressing a trigger button and simultaneously requesting the subject to start the task. The 30-s blocks for each condition were averaged with a 10-s baseline correction and a linear detrending. In more detail, we decided to average the different trials and then compute the FC of the hemodynamic courses, as this approach reduces the background noise.

### Data analysis

For data analysis MATLAB R2016b (MathWorks Inc., Natick, USA) was used. We corrected for high-amplitude movement artifacts through TDDR correction^58^ and the method of bandpass filtering (0.01–0.1 Hz) to filter out very high or low frequency artifacts; furthermore, a correlation-based signal improvement was performed to reduce motion influences on O2Hb-levels^59^. In further processing an independed component analysis (ICA)-based reduction of masticatory artifacts was performed. Artifact-loaded channels that outlasted preprocessing were visually detected and interpolated by surrounding channels. If more than 10% of the channels showed artifacts, the subject was excluded (in total n = 8 subjects). Finally, a gobal signal correction was performed by means of a PCA-based gaussian kernel filter^60^. The preprocessing steps were based on the guidelines of Brigadoi et al.^61^. 

For the computation of connectivity during task performance, data were averaged for each condition of the TMT. Furthermore, a baseline correction was performed where the activation during baseline measurement was subtracted from the activation during task completion. FC was calculated as Pearson correlation coefficients after the data of each channel pair was checked for multivariate outliers by malahanobis distances^37^. After FC indices were computed for each channel pair, FC between regions and within regions was computed by averaging the FC indices of the corresponding channels (e.g. all channels FCs of the left DLPFC to the right DLPFC).

### Statistics

After preprocessing, we compared the FCs within and between pre-defined region-specific nodes within the CCN and DAN: the somatosensory association cortex (SAC), dorsolateral prefrontal cortex (DLPFC) and inferior frontal gyrus (IFG). We computed FC for “within” and “between” region connections, either short-distance (ipsilateral) or long-distance, i.e. connections to contralateral regions^62^. Statistical data evaluation was performed with IBM SPSS Statistics Version 24.

We calculated repeated measures MANOVAs with the factors

- resting state and TMT (levels: RS, TMT-C, TMT-A, TMT-B; within-subjects),
- region of interest (ROI): 21 levels, each left (l), right (r):
  *either between:* lDLPFC_lIFG, lDLPFC_rIFG, lDLPFC_rDLPFC, lDLPFC_rSAC, lDLPFC_lSAC, rDLPFC_lIFG, rDLPFC_rIFG, rDLPFC_rSAC, rDLPFC_lSAC, lIFG_rIFG, lIFG_rSAC, lIFG_lSAC, rIFG_rSAC, rIFG_lSAC, rSAC_lSAC
  *or within:* rSAC_within, lSAC_within, lDLPFC_within, rDLPFC_within, lIFG_within, rIFG_within
  and
- age (< 65 and > 65 years; between-subjects).

Moreover, we investigated each ROI separately by a mixed ANOVA with the factors “task condition” (levels: RS, TMT-C, TMT-A, TMT-B; within-subjects) and “age” (< 65 and > 65 years; between-subjects).

Post-hoc analysis included simple contrasts (RS versus TMT-C, RS versus TMT-A, RS versus TMT-B) corrected by the Benjamini–Hochberg procedure, and Helmert contrasts (average TMT-A and TMT-B [TMT-A/B] vs TMT-C; TMT-A vs TMT-B).

## Results

### Behavioral results

#### General performance and age

Post-hoc analysis of Helmert contrasts confirmed fewer processed items during TMT-A/-B in comparison to TMT-C (*F*(1, 194) = 1227.80, *p* < 0.001, η^2^ = 0.86), fewer processed items during TMT-B in comparison to TMT-A (*F*(1, 194) = 1244.60, *p* < 0.001, η^2^ = 0.87) and fewer processed items in the late compared to the early elder subjects (Table 2).

**Table 2:** Demographics and behavioral data: Number of processed items during TMT-A, TMT-B and TMT-C, depending on age.

		Early elder Subjects (< 65 years)		Late elder Subjects (> 65 years)		*Statistics*
		Mean	*SD*	Mean	*SD*	
Age		60.23	2.98	70.27	4.46	–
TMT-A	Processed items	22.31	3.16	19.49	4.68	*p* < 0.001
TMT-B	Processed items	11.62	4.02	9.50	3.49	*p* < 0.001
TMT-C	Processed items	23.85	0.94	23.87	0.60	*p* = 0.86
TMT-B/-A	Processed items	0.53	0.18	0.51	0.26	*p* = 0.62

TMT, Trail Making Test; SD, standard deviation.

#### Interaction between age and task level

A two (age: early versus late elder subjects) by three (TMT: TMT-C versus TMT-A versus TMT-B) ANOVA showed a significant main effect of TMT (*F*(2, 388) = 1236.95, *p* < 0.001, η^2^ = 0.86) and a main effect of age (*F*(1, 194) = 25.81, *p* < 0.001, η^2^ = 0.12). Moreover, the interaction of age and TMT (*F*(2, 388) = 13.83, *p* < 0.001, η^2^ = 0.07) reflected that the age groups showed significant differences during TMT-A/B in comparison to TMT-C (*F*(1, 194) = 28.72, *p* < 0.001, η^2^ = 0.13) but not between TMT-A and TMT-B (*F*(1, 194) = 1.38, *p* = 0.241, η^2^ = 0.01).

The sexes did not differ in terms of the number of processed items (TMT-A: *t*(194) = − 1.25, *p* = 0.212, *d* = 0.20, TMT-B: *t*(194) = 0.16, *p* = 0.875, *d* = 0.02).

### fNIRS

#### Interaction between task level and ROI

**Figure 2:**
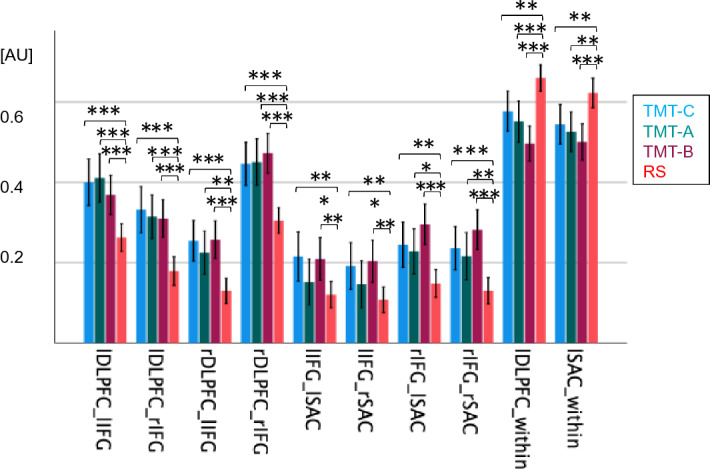
Mean between- (first eight bar groupings) and within-region (last two bar groupings) FC during RS versus the different TMT task conditions. Error bars indicate the 95% confidence interval. Asterisks represent statistically significant differences between RS and different task conditions for all the regions shown (* for *p* < 0.05, ** for *p* < 0.01 and *** for *p* < 0.001), see Table 3 as well as supplemental Table 1. RS, resting state; AU, arbitrary unit; TMT, Trail Making Test; DLPFC, dorsolateral prefrontal cortex; IFG, inferior frontal gyrus; SAC, sensory association cortex.

A repeated measures MANOVA revealed differences between the four measurement conditions (RS, TMT-C, TMT-A and TMT-B) concerning the ROI (*F*(63.00, 1678.40) = 8.24, Wilk’s Λ = 0.45, *p* < 0.001, partial η^2^ = 0.24). Concerning the FC within and between individual ROIs of the CCN and DAN, we observed multiple effects for the TMT. Between-regions differences were characterized by lower FC in RS than during TMT-A, -B and -C. In contrast, within-region effects were characterized by significantly higher FC at rest than during the TMT-A, -B and -C (Table 3, Fig. 2). For corresponding correlation matrices, please see our supplemental Figure 1.

**Table 3:** Between- and within-region FC in RS versus TMT task conditions.

	Statistics
**Between-region FC**	
lDLPFC_lIFG	*F* (2.74, 531.03) = 10.40 *p* < 0.001, η^2^ = 0.05
lDLPFC_rIFG	*F* (3, 582) = 11.06 *p* < 0.001, η^2^ = 0.05
rDLPFC_lIFG	*F* (2.87, 556.89) = 9.21 *p* < 0.001, η^2^ = 0.05
rDLPFC_rIFG	*F* (2.89, 560.38) = 13.56 *p* < 0.001, η^2^ = 0.07
lIFG_lSAC	*F* (3, 582) = 4.48 *p* = 0.042, η^2^ = 0.02
lIFG_rSAC	*F* (3, 582) = 4.31 *p* = 0.035, η^2^ = 0.02
rIFG_lSAC	*F* (3, 582) = 7.86 *p* < 0.001, η^2^ = 0.04
rIFG_rSAC	*F* (3, 582) = 9.26 *p* < 0.001, η^2^ = 0.05
**Within-region FC**	
lDLPFC	*F* (3, 582) = 13.08 *p* < 0.001, η^2^ = 0.06
lSAC	*F* (3, 582) = 7.23 *p* < 0.001, η^2^ = 0.04

FC, functional connectivity; DLPFC, dorsolateral prefrontal cortex; IFG, inferior frontal gyrus; SAC, sensory association cortex; l, left; r, right.

#### Interaction between age, task level and ROI.

**Figure 3:**
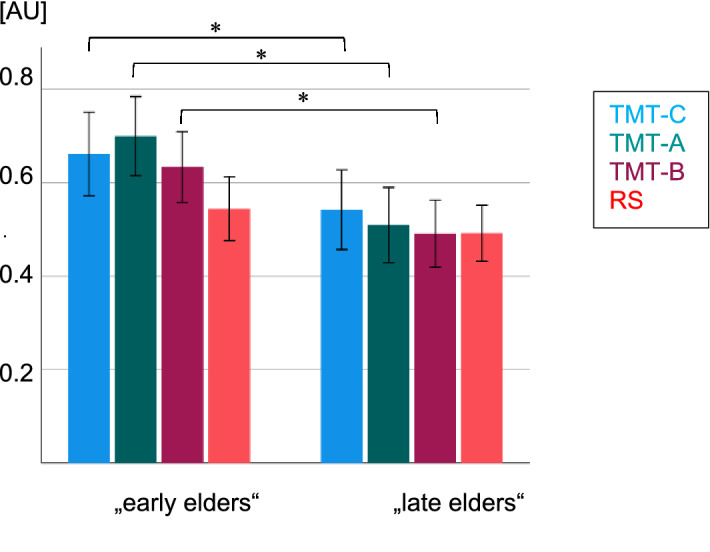
FC within the left IFG during RS versus the different TMT task conditions. “early elders”: participants < 65 years, “late elders”: participants > 65 years. Error bars indicate the 95% confidence interval. *Asterisks represent a statistically significant difference between the early and the late elder subgroup (p < 0.05). RS, resting state; TMT, Trail Making Test; AU, arbitrary unit.

Between-subjects, a main effect for age indicated lower FC within the ROI of the left IFG for the late as compared to the early elder subjects (lIFG_within: *F* (1, 194) = 10.17, *p* = 0.042, η^2^ = 0.05), independent of the corresponding condition (RS versus TMT). Moreover, we observed significant negative correlations of FC and age in various ROIs under the different TMT task conditions: During the TMT-A in the lIFG_within (*r* = − 0.22, p = 0.002) as well as between lIFG_rIFG (*r* = − 0.15, *p* = 0.040), during the TMT-B in the lIFG_within (*r* = − 0.16, *p* = 0.025) (Fig. 3) and during the TMT-C in the lDLPFC_within (*r* = − 0.14, *p* = 0.046).

## Discussion

The study at hand aimed to explore the differences in FC during TMT task completion and RS measurements depending on age (early versus late elder subjects) within a cohort at high risk for neurodegeneration. In summary, our results emphasize age-related task performance decline to be associated with changes of brain network organization, as it has on principle been described before^63^. Regarding the behavioral results, we generally confirmed fewer processed items during the TMT-A/-B in comparison to the TMT-C and fewer processed items during the TMT-B in comparison to the TMT-A. As expected, we found a reduced working speed with fewer processed items for the late elder as compared to the early elder subjects, which is in line with numerous previous studies^64–66^. In more detail, our results showed that this age-related difference in task performance was observed in comparison of the TMT-A/-B and TMT-C but not in comparison of the TMT-A and TMT-B. Therefore, it seems that the TMT-B, although being the cognitively more demanding task, might not be challenging enough to unmask age-related deficits earlier than the TMT-A. Alternatively, one could argue that the cognitive domain affected by age-related changes is indeed not primarily the executive function but rather general processing speed, though not simple motor velocity as both groups perform comparably at the TMT-C.

Most previous studies investigated age-related effects on FC during RS measurements^28,32,67–70^. Herefrom, it is a known phenomenon that in principle older compared to younger individuals show a higher between- as well as lower within-network FC during RS^28,31,36^, whereat Chan et al.^28^ even correlated this observation with reduced episodic memory scores across the adult lifespan. This seems to contradict our findings as we described a higher within- and lower between-network FC during RS within an older population, though, importantly, in comparison to a task performance condition. Moreover, a four-year, long-term MRI study by Ng et al.^33^ more precisely described the increase of between-network FC changes with advancing age to be preceded by an initial decrease. Fittingly, opposite effects for within-network FC, e.g. the DMN, have been described in the sense of an initial increase, followed by a later decrease^71,72^. It remains questionable whether this is a compensation mechanism that attempts to maintain the status quo with an initial hyper-recruitment. Within this context, it further has to be stated that the individuals investigated within previous studies were even older compared to our study cohort as a whole (e.g. Grady et al.^31^: mean 69.0 years) which is why one could argue they were situated at a different point on this timeline.

An aging-study on the comparison of FC during RS and TMT has not been reported yet, but there have been published approaches to compare FC between RS and other forms of task conditions, mainly in the context of fMRI studies. For instance, Di et al. and Cole et al.^73,74^ reported within-network connectivity to be decreased as well as between-network connectivity to be increased during task performance compared with RS, which is in line with our results. An MRI study by Arbabshirani et al.^75^ compared the functional network connectivity during RS and performance of an auditory oddball task. Their results during task performance, however, showed a global decrease of FC concomitant with an increased activity in particular networks. They suggested that successful performance of a certain task may be facilitated by an increased recruitment of related brain regions rather than collaboration among different networks, which, again, somehow contradicts our findings, whereat it is important to remark that the work of Arbabshirani et al.^75^ was not an aging study. With respect to FC, we identified discrepancies between brain regions depending on the mental state (RS versus TMT-C/-A/-B) as well. But interestingly, as described above, FC *within* regions of the CCN and DAN was higher at rest than during task performance, whereas FC *between* regions was higher during TMT performance compared to RS. Further results of a higher FC during RS in comparison to the task condition, similar to Arbabshirani et al.^75^, have already been reported by other authors^76–79^. Another possible explanation for this phenomenon might be the suppression of spontaneous thoughts by the attention demanding task^76^. Nevertheless, the current state of research is divided. Since other authors agreed on higher FC during task performance in comparison to RS measurements^80–82^, an influence of the cognitive task domain on FC magnitude and organization seems obvious. Actually, this has been described repeatedly within the fMRI studies of Varangis et al.^34,63^, e.g. in the sense of a higher age-related effect on FC measures during fluid reasoning compared to an episodic memory task. However, all these observations can serve to better classify the partly contradictory research results. Further, from a methodical point of view, one has to consider that the systematical performance of RS before the TMT task measurements may represent a confounding factor, even though the inverted order seems to be even more problematic, as in that case one would expect the participants to reflect during RS on their performance during the preceding task. Finally, an alternative view is provided by recent studies that suggest FC as a unique pattern that differs between individuals regardless of mental status including RS and task performance^83,84^.

When the variable age was taken into account within our investigation, a negative correlation between age and FC became visible in several ROIs; on principle, this was the case during both RS and the different forms of TMT performance, even though more pronounced under the task condition, as hypothesized. In this regard, we mainly found within-networks effects, namely within the left hemisphere for the IFG and DLPFC. Furthermore, bilateral connectivity between the right and left IFG also was reduced. Hence, it seems that mainly reduced within-network FC, especially in the ROI of the left IFG, is associated with a reduced cognitive performance in advanced age. Interestingly, for the specific regions in which an age effect showed up within our investigation, other authors even described a regional hypometabolism in FDG-PET in the context of different forms of early dementia and subsequent especially executive deficits^85^. We consciously selected our aging cohort on the whole to exhibit even other high-risk factors for neurodegeneration (e.g. RBD and/or depression). Importantly, the aim of this approach was not to investigate the influence of single neurodegenerative risk factors apart from age on FC. Instead, we intended to increase the probability of the overall study cohort to already present (age-related) neurodegenerative changes influencing FC measurements. Notably, only the FC between the left IFG and right SAC was positively correlated with age in our investigation. A generally increased cortical activity measured via fNIRS in older participants during performance of the TMT has already been described by us as well as discussed within the context of a possible compensation mechanism^86^. For instance, Respino et al.^25^ reported a positive correlation between an elevated regional homogeneity within the dACC as part of the CCN and executive performance in the context of late-life depression and suggested this to be a possible compensation mechanism, too.

Some limitations of our study should also be mentioned. First, the used neuroimaging method of fNIRS has established itself as a reliable alternative to fMRI in FC studies because it combines an easy clinical integration with ecologically valid conditions (realistic environment, sitting position, task accomplishment, social interaction) as well as relatively high time resolution^38^. But unlike MRI-based technology, fNIRS does not allow to put functional and structural information into relation; this is important to note as, on the one hand, it is well known that particularly at early stages of a neurodegenerative disease the correlation between cognitive dysfunction and structural gray or white matter changes can by no means be presumed^87,88^; on the other hand, a relevant cortical atrophy especially in fronto-temporal areas has been described even in elderly with low probability of AD^1^. Further, due to its shallow penetration depth of 2–3 cm into the cranial calotte^89^, only superficial cortical structures are measured, whereas deeper connections to and within white matter structures will not be captured; this might be a disadvantage within the context of dementia entities with relevant subcortical involvement of pathology, e.g. Parkinson’s Disease or vascular dementia. Finally, albeit the CCN and DAN have already been shown to be accessible for fNIRS^26^, subsequent analysis is also limited to the corresponding pre-defined ROIs. Besides, the spatial specificity of fNIRS compared to fMRI is reduced, which means that measuring FC within a region by correlation techniques can overestimate the real FC because the individual channels may access overlapping areas. Anyway, the combination of fNIRS with the TMT has already been shown to be suitable for the investigation of elderly as well as the detection of aging-related differences in resulting cortical activation patterns^2,43,90,91^. The TMT allows the integration into many clinical study settings because it is an easy-to-handle paper–pencil task that offers a natural testing situation and does not provoke any artifacts by activating the mimic musculature by speech.

Next, our subject sample shows an age range of 50 to 85 years as FC changes during later life as well as associated executive deficits were the main focus of the study at hand. So, one could argue that the omission of younger volunteers prevents a complete picture of FC changes over the lifespan, although previous studies have already shown that at least age-related cognitive deficits do not become apparent until about 50 years of age^92–95^. Accordingly, one might assume that even the concomitant FC changes during task performance are not expected to appear before that age, either. However, Hofmann et al.^43^ observed a significantly reduced neural activity in the right DLPFC also via fNIRS within prodromal Parkinson’s Disease patients completing the TMT-A and -B in contrast to the TMT-C, even before differences became evident on the behavioral level.

Finally, as described above, we investigated a sub-cohort of the TREND study collective, which is enriched for other neurodegenerative risk factors apart from age. This is why we matched the two groups of early and late elders, amongst others, for these factors (Table 1). Even though they should thus be equally present in both subgroups, this may bias our results, nevertheless. Therefore, within future studies, it will be important to potentially create a cohort of healthy agers explicitly free from other neurodegenerative risk factors or enriched for only single of these risk factors. Such an approach might help to selectively investigate the influence of other risk factors for neurodegenerative diseases, like RBD, on FC during the prodromal stage.

## Conclusion

To the authors’ knowledge, this is the first study examining the influence of age on FC through a comparison of RS and task-related measurements (TMT) using fNIRS.

To sum up, only particular regions of the CCN and DAN were affected by an age-related FC decrease. This finding was observed for the RS measurement and was even more pronounced during execution of the TMT, and it might indicate a specific vulnerability of these areas to aging and/or early neurodegenerative processes. Further, with the aim of identifying age-related—either physiological or pathological—FC changes as early as possible, these results confirm our hypothesis that measuring these during task conditions is superior to the RS.

Therefore, it will be very important to confirm these promising findings within future studies, under special consideration of multiple task conditions on FC characteristics of the corresponding networks. Especially the application of a dual task situation might be a promising approach to further understand this dynamic and complex interplay. For instance, Beurskens et al.^96^ have already shown within their fNIRS study that especially the combination of a cognitive with a simultaneous motor task can have a relevant impact on neural functionality within older adults. Finally, the constant correlation with behavioral performance will maintain a key role for assessment of potentially successful, neural compensation mechanisms.

## Supplementary Information

Supplementary Information.

## Data Availability

The datasets generated and/or analyzed during this study are available by request.
