# Supplementary material for: Effects of aging on functional connectivity in a neurodegenerative risk cohort: resting state versus task measurement using near-infrared spectroscopy
Source: Sci Rep. 2022 Jul 4;12:11262. doi: 10.1038/s41598-022-13326-7 (PMC9253312; doi:10.1038/s41598-022-13326-7)
Supplement: Supplementary file 1 — Supplementary Information. [file 41598_2022_13326_MOESM1_ESM.docx]

**Supplements**


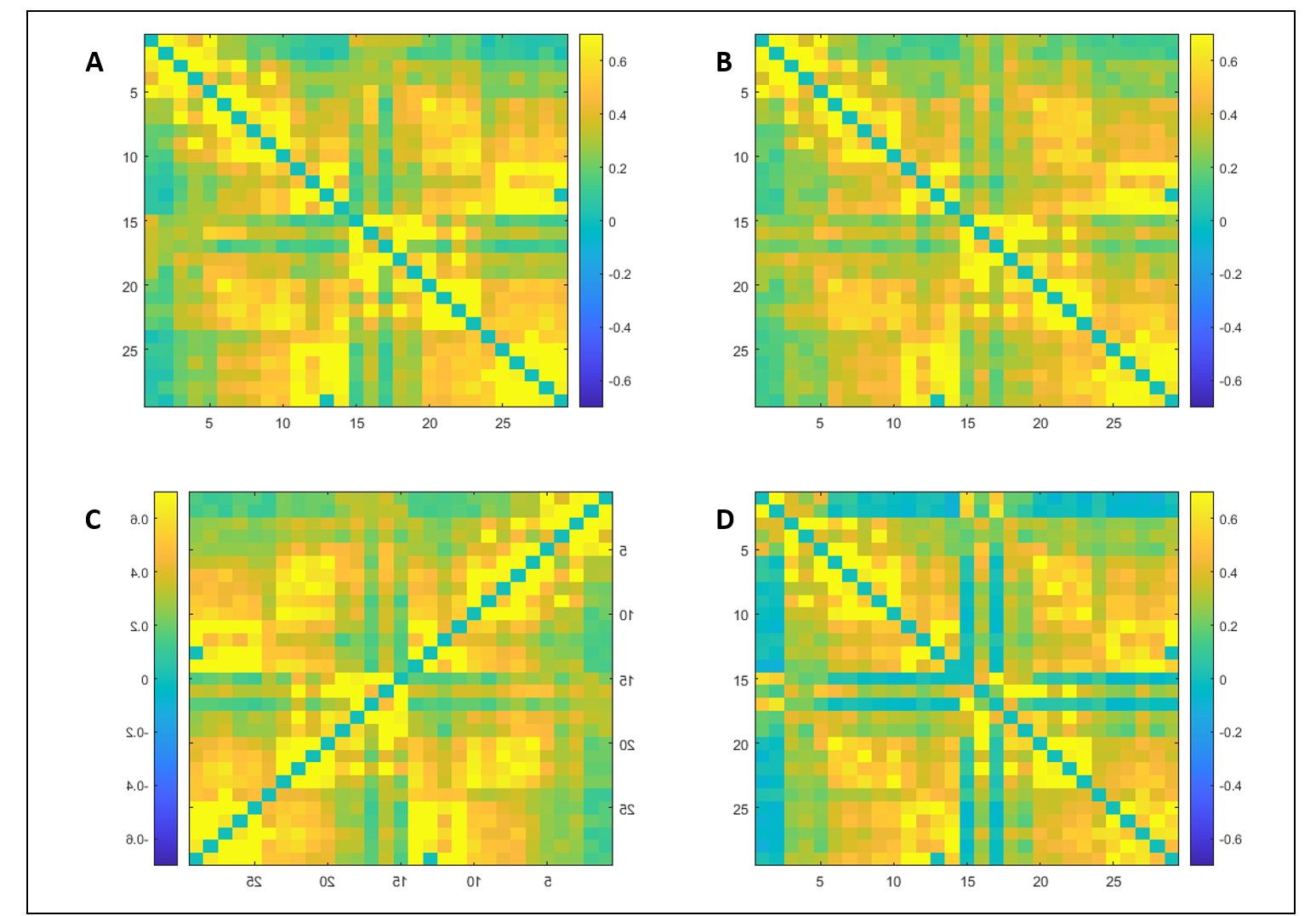


**Supplemental figure 1:** Correlation matrices across all the regions investigated. A: TMT-A, B: TMT-B, C: TMT-C, D: resting state. The different ROIs are indicated via the following rows/columns: 1/4: lIFG, 5/11: lDLPFC, 12/15: lSAC, 16/19: rIFG, 20/25: rDLPFC, 26/30: rSAC.

|  | | | | | | | | |
| --- | --- | --- | --- | --- | --- | --- | --- | --- |
| Source | Measure | TMT | Sum of squares | df | Mean of squares | F | Level of significance | Partial Eta-square |
| TMT | ldlpfc_lifg | Niveau 1 vs. Niveau 4 | 3,732 | 1 | 3,732 | 17,460 | ,000 | ,083 |
|  |  | Niveau 2 vs. Niveau 4 | 4,304 | 1 | 4,304 | 18,846 | ,000 | ,089 |
|  |  | Niveau 3 vs. Niveau 4 | 2,226 | 1 | 2,226 | 12,586 | ,000 | ,061 |
|  | ldlpfc_rifg | Niveau 1 vs. Niveau 4 | 4,537 | 1 | 4,537 | 24,515 | ,000 | ,112 |
|  |  | Niveau 2 vs. Niveau 4 | 3,580 | 1 | 3,580 | 18,963 | ,000 | ,089 |
|  |  | Niveau 3 vs. Niveau 4 | 3,354 | 1 | 3,354 | 19,048 | ,000 | ,089 |
|  | ldlpfc_rdlpfc | Niveau 1 vs. Niveau 4 | 1,223 | 1 | 1,223 | 9,692 | ,002 | ,048 |
|  |  | Niveau 2 vs. Niveau 4 | ,194 | 1 | ,194 | 1,280 | ,259 | ,007 |
|  |  | Niveau 3 vs. Niveau 4 | ,094 | 1 | ,094 | ,754 | ,386 | ,004 |
|  | ldlpfc_rsac | Niveau 1 vs. Niveau 4 | ,014 | 1 | ,014 | ,102 | ,750 | ,001 |
|  |  | Niveau 2 vs. Niveau 4 | ,009 | 1 | ,009 | ,059 | ,808 | ,000 |
|  |  | Niveau 3 vs. Niveau 4 | ,114 | 1 | ,114 | 1,096 | ,297 | ,006 |
|  | ldlpfc_lsac | Niveau 1 vs. Niveau 4 | ,002 | 1 | ,002 | ,015 | ,902 | ,000 |
|  |  | Niveau 2 vs. Niveau 4 | ,087 | 1 | ,087 | ,546 | ,461 | ,003 |
|  |  | Niveau 3 vs. Niveau 4 | ,153 | 1 | ,153 | 1,386 | ,241 | ,007 |
|  | ldlpfc_within | Niveau 1 vs. Niveau 4 | 1,342 | 1 | 1,342 | 8,572 | ,004 | ,042 |
|  |  | Niveau 2 vs. Niveau 4 | 2,311 | 1 | 2,311 | 14,265 | ,000 | ,068 |
|  |  | Niveau 3 vs. Niveau 4 | 5,219 | 1 | 5,219 | 41,317 | ,000 | ,176 |
|  | rdlpfc_lifg | Niveau 1 vs. Niveau 4 | 3,049 | 1 | 3,049 | 18,409 | ,000 | ,087 |
|  |  | Niveau 2 vs. Niveau 4 | 1,782 | 1 | 1,782 | 9,857 | ,002 | ,048 |
|  |  | Niveau 3 vs. Niveau 4 | 3,195 | 1 | 3,195 | 21,360 | ,000 | ,099 |
|  | rdlpfc_rifg | Niveau 1 vs. Niveau 4 | 3,903 | 1 | 3,903 | 22,454 | ,000 | ,104 |
|  |  | Niveau 2 vs. Niveau 4 | 4,169 | 1 | 4,169 | 19,747 | ,000 | ,092 |
|  |  | Niveau 3 vs. Niveau 4 | 5,489 | 1 | 5,489 | 33,246 | ,000 | ,146 |
|  | rdlpfc_rsac | Niveau 1 vs. Niveau 4 | ,933 | 1 | ,933 | 6,660 | ,011 | ,033 |
|  |  | Niveau 2 vs. Niveau 4 | ,943 | 1 | ,943 | 6,304 | ,013 | ,031 |
|  |  | Niveau 3 vs. Niveau 4 | ,595 | 1 | ,595 | 5,507 | ,020 | ,028 |
|  | rdlpfc_lsac | Niveau 1 vs. Niveau 4 | ,411 | 1 | ,411 | 2,822 | ,095 | ,014 |
|  |  | Niveau 2 vs. Niveau 4 | ,530 | 1 | ,530 | 3,434 | ,065 | ,017 |
|  |  | Niveau 3 vs. Niveau 4 | ,321 | 1 | ,321 | 2,743 | ,099 | ,014 |
|  | rdlpfc_within | Niveau 1 vs. Niveau 4 | ,563 | 1 | ,563 | 4,686 | ,032 | ,024 |
|  |  | Niveau 2 vs. Niveau 4 | ,394 | 1 | ,394 | 3,032 | ,083 | ,015 |
|  |  | Niveau 3 vs. Niveau 4 | ,735 | 1 | ,735 | 6,225 | ,013 | ,031 |
|  | lifg_rifg | Niveau 1 vs. Niveau 4 | ,011 | 1 | ,011 | ,054 | ,816 | ,000 |
|  |  | Niveau 2 vs. Niveau 4 | ,098 | 1 | ,098 | ,455 | ,501 | ,002 |
|  |  | Niveau 3 vs. Niveau 4 | ,141 | 1 | ,141 | ,850 | ,358 | ,004 |
|  | lifg_rsac | Niveau 1 vs. Niveau 4 | 1,401 | 1 | 1,401 | 7,357 | ,007 | ,037 |
|  |  | Niveau 2 vs. Niveau 4 | ,290 | 1 | ,290 | 1,438 | ,232 | ,007 |
|  |  | Niveau 3 vs. Niveau 4 | 1,812 | 1 | 1,812 | 10,251 | ,002 | ,050 |
|  | lifg_lsac | Niveau 1 vs. Niveau 4 | 1,780 | 1 | 1,780 | 8,425 | ,004 | ,042 |
|  |  | Niveau 2 vs. Niveau 4 | ,195 | 1 | ,195 | ,980 | ,323 | ,005 |
|  |  | Niveau 3 vs. Niveau 4 | 1,558 | 1 | 1,558 | 8,422 | ,004 | ,042 |
|  | lifg_within | Niveau 1 vs. Niveau 4 | 1,367 | 1 | 1,367 | 5,273 | ,023 | ,026 |
|  |  | Niveau 2 vs. Niveau 4 | 1,458 | 1 | 1,458 | 5,804 | ,017 | ,029 |
|  |  | Niveau 3 vs. Niveau 4 | ,382 | 1 | ,382 | 1,620 | ,205 | ,008 |
|  | rifg_rsac | Niveau 1 vs. Niveau 4 | 2,209 | 1 | 2,209 | 12,980 | ,000 | ,063 |
|  |  | Niveau 2 vs. Niveau 4 | 1,451 | 1 | 1,451 | 7,007 | ,009 | ,035 |
|  |  | Niveau 3 vs. Niveau 4 | 4,540 | 1 | 4,540 | 28,412 | ,000 | ,128 |
|  | rifg_lsac | Niveau 1 vs. Niveau 4 | 1,821 | 1 | 1,821 | 9,112 | ,003 | ,045 |
|  |  | Niveau 2 vs. Niveau 4 | 1,253 | 1 | 1,253 | 5,891 | ,016 | ,029 |
|  |  | Niveau 3 vs. Niveau 4 | 4,242 | 1 | 4,242 | 24,472 | ,000 | ,112 |
|  | rifg_within | Niveau 1 vs. Niveau 4 | ,391 | 1 | ,391 | 1,520 | ,219 | ,008 |
|  |  | Niveau 2 vs. Niveau 4 | ,003 | 1 | ,003 | ,014 | ,905 | ,000 |
|  |  | Niveau 3 vs. Niveau 4 | ,265 | 1 | ,265 | 1,095 | ,297 | ,006 |
|  | rsac_lsac | Niveau 1 vs. Niveau 4 | ,721 | 1 | ,721 | 4,124 | ,044 | ,021 |
|  |  | Niveau 2 vs. Niveau 4 | ,678 | 1 | ,678 | 4,255 | ,040 | ,021 |
|  |  | Niveau 3 vs. Niveau 4 | ,058 | 1 | ,058 | ,419 | ,518 | ,002 |
|  | rsac_within | Niveau 1 vs. Niveau 4 | ,536 | 1 | ,536 | 3,364 | ,068 | ,017 |
|  |  | Niveau 2 vs. Niveau 4 | ,360 | 1 | ,360 | 2,521 | ,114 | ,013 |
|  |  | Niveau 3 vs. Niveau 4 | ,879 | 1 | ,879 | 6,559 | ,011 | ,033 |
|  | lsac_within | Niveau 1 vs. Niveau 4 | 1,188 | 1 | 1,188 | 7,343 | ,007 | ,036 |
|  |  | Niveau 2 vs. Niveau 4 | 1,824 | 1 | 1,824 | 11,359 | ,001 | ,055 |
|  |  | Niveau 3 vs. Niveau 4 | 2,915 | 1 | 2,915 | 19,097 | ,000 | ,090 |
| TMT * age | ldlpfc_lifg | Niveau 1 vs. Niveau 4 | ,015 | 1 | ,015 | ,070 | ,791 | ,000 |
|  |  | Niveau 2 vs. Niveau 4 | ,058 | 1 | ,058 | ,255 | ,614 | ,001 |
|  |  | Niveau 3 vs. Niveau 4 | ,112 | 1 | ,112 | ,632 | ,428 | ,003 |
|  | ldlpfc_rifg | Niveau 1 vs. Niveau 4 | ,047 | 1 | ,047 | ,255 | ,614 | ,001 |
|  |  | Niveau 2 vs. Niveau 4 | ,364 | 1 | ,364 | 1,930 | ,166 | ,010 |
|  |  | Niveau 3 vs. Niveau 4 | ,002 | 1 | ,002 | ,011 | ,916 | ,000 |
|  | ldlpfc_rdlpfc | Niveau 1 vs. Niveau 4 | ,013 | 1 | ,013 | ,104 | ,748 | ,001 |
|  |  | Niveau 2 vs. Niveau 4 | ,000 | 1 | ,000 | ,001 | ,969 | ,000 |
|  |  | Niveau 3 vs. Niveau 4 | 6,177E-6 | 1 | 6,177E-6 | ,000 | ,994 | ,000 |
|  | ldlpfc_rsac | Niveau 1 vs. Niveau 4 | ,099 | 1 | ,099 | ,719 | ,398 | ,004 |
|  |  | Niveau 2 vs. Niveau 4 | ,001 | 1 | ,001 | ,008 | ,929 | ,000 |
|  |  | Niveau 3 vs. Niveau 4 | ,005 | 1 | ,005 | ,052 | ,820 | ,000 |
|  | ldlpfc_lsac | Niveau 1 vs. Niveau 4 | ,356 | 1 | ,356 | 2,299 | ,131 | ,012 |
|  |  | Niveau 2 vs. Niveau 4 | ,008 | 1 | ,008 | ,051 | ,821 | ,000 |
|  |  | Niveau 3 vs. Niveau 4 | ,001 | 1 | ,001 | ,005 | ,942 | ,000 |
|  | ldlpfc_within | Niveau 1 vs. Niveau 4 | ,092 | 1 | ,092 | ,590 | ,443 | ,003 |
|  |  | Niveau 2 vs. Niveau 4 | ,082 | 1 | ,082 | ,506 | ,478 | ,003 |
|  |  | Niveau 3 vs. Niveau 4 | ,121 | 1 | ,121 | ,959 | ,329 | ,005 |
|  | rdlpfc_lifg | Niveau 1 vs. Niveau 4 | ,141 | 1 | ,141 | ,853 | ,357 | ,004 |
|  |  | Niveau 2 vs. Niveau 4 | ,241 | 1 | ,241 | 1,335 | ,249 | ,007 |
|  |  | Niveau 3 vs. Niveau 4 | ,231 | 1 | ,231 | 1,542 | ,216 | ,008 |
|  | rdlpfc_rifg | Niveau 1 vs. Niveau 4 | ,028 | 1 | ,028 | ,162 | ,688 | ,001 |
|  |  | Niveau 2 vs. Niveau 4 | ,017 | 1 | ,017 | ,080 | ,777 | ,000 |
|  |  | Niveau 3 vs. Niveau 4 | ,005 | 1 | ,005 | ,031 | ,861 | ,000 |
|  | rdlpfc_rsac | Niveau 1 vs. Niveau 4 | ,525 | 1 | ,525 | 3,745 | ,054 | ,019 |
|  |  | Niveau 2 vs. Niveau 4 | ,058 | 1 | ,058 | ,389 | ,534 | ,002 |
|  |  | Niveau 3 vs. Niveau 4 | ,318 | 1 | ,318 | 2,946 | ,088 | ,015 |
|  | rdlpfc_lsac | Niveau 1 vs. Niveau 4 | ,268 | 1 | ,268 | 1,841 | ,176 | ,009 |
|  |  | Niveau 2 vs. Niveau 4 | ,112 | 1 | ,112 | ,727 | ,395 | ,004 |
|  |  | Niveau 3 vs. Niveau 4 | ,628 | 1 | ,628 | 5,358 | ,022 | ,027 |
|  | rdlpfc_within | Niveau 1 vs. Niveau 4 | ,275 | 1 | ,275 | 2,289 | ,132 | ,012 |
|  |  | Niveau 2 vs. Niveau 4 | ,172 | 1 | ,172 | 1,323 | ,252 | ,007 |
|  |  | Niveau 3 vs. Niveau 4 | ,041 | 1 | ,041 | ,344 | ,558 | ,002 |
|  | lifg_rifg | Niveau 1 vs. Niveau 4 | ,136 | 1 | ,136 | ,659 | ,418 | ,003 |
|  |  | Niveau 2 vs. Niveau 4 | ,130 | 1 | ,130 | ,607 | ,437 | ,003 |
|  |  | Niveau 3 vs. Niveau 4 | ,053 | 1 | ,053 | ,319 | ,573 | ,002 |
|  | lifg_rsac | Niveau 1 vs. Niveau 4 | ,399 | 1 | ,399 | 2,097 | ,149 | ,011 |
|  |  | Niveau 2 vs. Niveau 4 | ,001 | 1 | ,001 | ,005 | ,944 | ,000 |
|  |  | Niveau 3 vs. Niveau 4 | ,217 | 1 | ,217 | 1,225 | ,270 | ,006 |
|  | lifg_lsac | Niveau 1 vs. Niveau 4 | ,261 | 1 | ,261 | 1,237 | ,267 | ,006 |
|  |  | Niveau 2 vs. Niveau 4 | ,007 | 1 | ,007 | ,037 | ,848 | ,000 |
|  |  | Niveau 3 vs. Niveau 4 | ,680 | 1 | ,680 | 3,676 | ,057 | ,019 |
|  | lifg_within | Niveau 1 vs. Niveau 4 | ,217 | 1 | ,217 | ,837 | ,361 | ,004 |
|  |  | Niveau 2 vs. Niveau 4 | ,922 | 1 | ,922 | 3,672 | ,057 | ,019 |
|  |  | Niveau 3 vs. Niveau 4 | ,399 | 1 | ,399 | 1,693 | ,195 | ,009 |
|  | rifg_rsac | Niveau 1 vs. Niveau 4 | ,154 | 1 | ,154 | ,906 | ,342 | ,005 |
|  |  | Niveau 2 vs. Niveau 4 | ,169 | 1 | ,169 | ,815 | ,368 | ,004 |
|  |  | Niveau 3 vs. Niveau 4 | ,016 | 1 | ,016 | ,102 | ,749 | ,001 |
|  | rifg_lsac | Niveau 1 vs. Niveau 4 | ,002 | 1 | ,002 | ,012 | ,912 | ,000 |
|  |  | Niveau 2 vs. Niveau 4 | ,311 | 1 | ,311 | 1,463 | ,228 | ,007 |
|  |  | Niveau 3 vs. Niveau 4 | ,199 | 1 | ,199 | 1,150 | ,285 | ,006 |
|  | rifg_within | Niveau 1 vs. Niveau 4 | ,039 | 1 | ,039 | ,153 | ,696 | ,001 |
|  |  | Niveau 2 vs. Niveau 4 | ,078 | 1 | ,078 | ,353 | ,553 | ,002 |
|  |  | Niveau 3 vs. Niveau 4 | ,004 | 1 | ,004 | ,017 | ,896 | ,000 |
|  | rsac_lsac | Niveau 1 vs. Niveau 4 | ,432 | 1 | ,432 | 2,472 | ,118 | ,013 |
|  |  | Niveau 2 vs. Niveau 4 | ,126 | 1 | ,126 | ,788 | ,376 | ,004 |
|  |  | Niveau 3 vs. Niveau 4 | ,386 | 1 | ,386 | 2,768 | ,098 | ,014 |
|  | rsac_within | Niveau 1 vs. Niveau 4 | ,281 | 1 | ,281 | 1,766 | ,185 | ,009 |
|  |  | Niveau 2 vs. Niveau 4 | ,201 | 1 | ,201 | 1,411 | ,236 | ,007 |
|  |  | Niveau 3 vs. Niveau 4 | ,421 | 1 | ,421 | 3,140 | ,078 | ,016 |
|  | lsac_within | Niveau 1 vs. Niveau 4 | ,537 | 1 | ,537 | 3,321 | ,070 | ,017 |
|  |  | Niveau 2 vs. Niveau 4 | ,366 | 1 | ,366 | 2,282 | ,132 | ,012 |
|  |  | Niveau 3 vs. Niveau 4 | ,196 | 1 | ,196 | 1,281 | ,259 | ,007 |
| Error (TMT) | ldlpfc_lifg | Niveau 1 vs. Niveau 4 | 41,472 | 194 | ,214 |  |  |  |
|  |  | Niveau 2 vs. Niveau 4 | 44,302 | 194 | ,228 |  |  |  |
|  |  | Niveau 3 vs. Niveau 4 | 34,305 | 194 | ,177 |  |  |  |
|  | ldlpfc_rifg | Niveau 1 vs. Niveau 4 | 35,901 | 194 | ,185 |  |  |  |
|  |  | Niveau 2 vs. Niveau 4 | 36,622 | 194 | ,189 |  |  |  |
|  |  | Niveau 3 vs. Niveau 4 | 34,162 | 194 | ,176 |  |  |  |
|  | ldlpfc_rdlpfc | Niveau 1 vs. Niveau 4 | 24,484 | 194 | ,126 |  |  |  |
|  |  | Niveau 2 vs. Niveau 4 | 29,350 | 194 | ,151 |  |  |  |
|  |  | Niveau 3 vs. Niveau 4 | 24,174 | 194 | ,125 |  |  |  |
|  | ldlpfc_rsac | Niveau 1 vs. Niveau 4 | 26,660 | 194 | ,137 |  |  |  |
|  |  | Niveau 2 vs. Niveau 4 | 30,278 | 194 | ,156 |  |  |  |
|  |  | Niveau 3 vs. Niveau 4 | 20,252 | 194 | ,104 |  |  |  |
|  | ldlpfc_lsac | Niveau 1 vs. Niveau 4 | 30,028 | 194 | ,155 |  |  |  |
|  |  | Niveau 2 vs. Niveau 4 | 30,939 | 194 | ,159 |  |  |  |
|  |  | Niveau 3 vs. Niveau 4 | 21,395 | 194 | ,110 |  |  |  |
|  | ldlpfc_within | Niveau 1 vs. Niveau 4 | 30,375 | 194 | ,157 |  |  |  |
|  |  | Niveau 2 vs. Niveau 4 | 31,434 | 194 | ,162 |  |  |  |
|  |  | Niveau 3 vs. Niveau 4 | 24,504 | 194 | ,126 |  |  |  |
|  | rdlpfc_lifg | Niveau 1 vs. Niveau 4 | 32,130 | 194 | ,166 |  |  |  |
|  |  | Niveau 2 vs. Niveau 4 | 35,076 | 194 | ,181 |  |  |  |
|  |  | Niveau 3 vs. Niveau 4 | 29,016 | 194 | ,150 |  |  |  |
|  | rdlpfc_rifg | Niveau 1 vs. Niveau 4 | 33,719 | 194 | ,174 |  |  |  |
|  |  | Niveau 2 vs. Niveau 4 | 40,960 | 194 | ,211 |  |  |  |
|  |  | Niveau 3 vs. Niveau 4 | 32,028 | 194 | ,165 |  |  |  |
|  | rdlpfc_rsac | Niveau 1 vs. Niveau 4 | 27,178 | 194 | ,140 |  |  |  |
|  |  | Niveau 2 vs. Niveau 4 | 29,023 | 194 | ,150 |  |  |  |
|  |  | Niveau 3 vs. Niveau 4 | 20,972 | 194 | ,108 |  |  |  |
|  | rdlpfc_lsac | Niveau 1 vs. Niveau 4 | 28,241 | 194 | ,146 |  |  |  |
|  |  | Niveau 2 vs. Niveau 4 | 29,962 | 194 | ,154 |  |  |  |
|  |  | Niveau 3 vs. Niveau 4 | 22,731 | 194 | ,117 |  |  |  |
|  | rdlpfc_within | Niveau 1 vs. Niveau 4 | 23,289 | 194 | ,120 |  |  |  |
|  |  | Niveau 2 vs. Niveau 4 | 25,194 | 194 | ,130 |  |  |  |
|  |  | Niveau 3 vs. Niveau 4 | 22,908 | 194 | ,118 |  |  |  |
|  | lifg_rifg | Niveau 1 vs. Niveau 4 | 39,947 | 194 | ,206 |  |  |  |
|  |  | Niveau 2 vs. Niveau 4 | 41,597 | 194 | ,214 |  |  |  |
|  |  | Niveau 3 vs. Niveau 4 | 32,254 | 194 | ,166 |  |  |  |
|  | lifg_rsac | Niveau 1 vs. Niveau 4 | 36,944 | 194 | ,190 |  |  |  |
|  |  | Niveau 2 vs. Niveau 4 | 39,120 | 194 | ,202 |  |  |  |
|  |  | Niveau 3 vs. Niveau 4 | 34,296 | 194 | ,177 |  |  |  |
|  | lifg_lsac | Niveau 1 vs. Niveau 4 | 40,978 | 194 | ,211 |  |  |  |
|  |  | Niveau 2 vs. Niveau 4 | 38,506 | 194 | ,198 |  |  |  |
|  |  | Niveau 3 vs. Niveau 4 | 35,890 | 194 | ,185 |  |  |  |
|  | lifg_within | Niveau 1 vs. Niveau 4 | 50,292 | 194 | ,259 |  |  |  |
|  |  | Niveau 2 vs. Niveau 4 | 48,741 | 194 | ,251 |  |  |  |
|  |  | Niveau 3 vs. Niveau 4 | 45,724 | 194 | ,236 |  |  |  |
|  | rifg_rsac | Niveau 1 vs. Niveau 4 | 33,018 | 194 | ,170 |  |  |  |
|  |  | Niveau 2 vs. Niveau 4 | 40,169 | 194 | ,207 |  |  |  |
|  |  | Niveau 3 vs. Niveau 4 | 30,997 | 194 | ,160 |  |  |  |
|  | rifg_lsac | Niveau 1 vs. Niveau 4 | 38,763 | 194 | ,200 |  |  |  |
|  |  | Niveau 2 vs. Niveau 4 | 41,247 | 194 | ,213 |  |  |  |
|  |  | Niveau 3 vs. Niveau 4 | 33,629 | 194 | ,173 |  |  |  |
|  | rifg_within | Niveau 1 vs. Niveau 4 | 49,866 | 194 | ,257 |  |  |  |
|  |  | Niveau 2 vs. Niveau 4 | 42,829 | 194 | ,221 |  |  |  |
|  |  | Niveau 3 vs. Niveau 4 | 46,922 | 194 | ,242 |  |  |  |
|  | rsac_lsac | Niveau 1 vs. Niveau 4 | 33,915 | 194 | ,175 |  |  |  |
|  |  | Niveau 2 vs. Niveau 4 | 30,910 | 194 | ,159 |  |  |  |
|  |  | Niveau 3 vs. Niveau 4 | 27,089 | 194 | ,140 |  |  |  |
|  | rsac_within | Niveau 1 vs. Niveau 4 | 30,901 | 194 | ,159 |  |  |  |
|  |  | Niveau 2 vs. Niveau 4 | 27,706 | 194 | ,143 |  |  |  |
|  |  | Niveau 3 vs. Niveau 4 | 26,007 | 194 | ,134 |  |  |  |
|  | lsac_within | Niveau 1 vs. Niveau 4 | 31,392 | 194 | ,162 |  |  |  |
|  |  | Niveau 2 vs. Niveau 4 | 31,143 | 194 | ,161 |  |  |  |
|  |  | Niveau 3 vs. Niveau 4 | 29,616 | 194 | ,153 |  |  |  |

**Supplemental table 1:** Level of significance for different ROI activation patterns in fNIRS according to the task condition: *Niveau 1* = TMT-C, *Niveau 2* = TMT-A, *Niveau 3* = TMT-B, *Niveau 4* = resting state, DLPFC = dorso-lateral prefrontal cortex, IFG = inferior frontal gyrus, SAC = sensory association cortex.
